# Autophagy promotes p72 degradation and capsid disassembly during the early phase of African swine fever virus infection

**DOI:** 10.1128/jvi.01701-24

**Published:** 2024-12-17

**Authors:** Jie Song, Jiangnan Li, Shuai Li, Gaihong Zhao, Tingting Li, Xin Chen, Boli Hu, Jia Liu, Xinyu Lai, Sitong Liu, Qiongqiong Zhou, Li Huang, Changjiang Weng

**Affiliations:** 1 Division of Fundamental Immunology, National African Swine Fever Para-reference Laboratory, State Key Laboratory for Animal Disease Control and Prevention, Harbin Veterinary Research Institute, Chinese Academy of Agricultural Sciences 687216 , Harbin, Heilongjiang, China; 2 Heilongjiang Provincial Key Laboratory of Veterinary Immunology, Harbin, China; 3 MOA Key Laboratory of Animal Virology, Center for Veterinary Sciences, Zhejiang University 12377 , Hangzhou, Zhejiang, China; The University of Arizona, Tucson, Arizona, USA

**Keywords:** African swine fever virus, p72, selective autophagy, Stub1, HSPA8, capsid disassembly

## Abstract

During viral infections, autophagy functions as a cell-intrinsic defense mechanism by facilitating the delivery of virions or viral components to the endosomal/lysosomal pathway for degradation. In this study, we report that internalized African swine fever virus (ASFV) virions enter autolysosomes during the early phase of viral infection. Autophagy selectively targets the major capsid protein p72 within the ASFV virion. The ASFV p72 protein undergoes modification through ubiquitination at the C-terminus, a process mediated by the E3 ubiquitin ligase Stub1. Subsequently, ubiquitinated p72 is recognized by the autophagy receptor SQSTM1/p62 through its ubiquitin-binding domain. Stub1 facilitates the ubiquitination and degradation of p72 in an HSPA8-dependent manner *via* selective autophagy. Autophagy plays a critical role in disassembling ASFV virions and further promotes the release of ASFV genomic DNA. These findings support the notion that autophagy is involved in and contributes to the capsid disassembly of ASFV, providing valuable insights into this essential viral process.

**IMPORTANCE:**

African swine fever (ASF), a highly contagious disease caused by the ASF virus (ASFV), affects domestic pigs and wild boars, with a mortality rate of up to 100%. The ASF epidemic poses a persistent threat to the global pig industry. Currently, no effective vaccines or antiviral drugs are available for prevention and control. In this study, we discovered that autophagy promotes the degradation of p72 and the disassembly of the capsid during the early phase of ASFV infection. Mechanically, Stub1 facilitates the polyubiquitination of ASFV p72 through the chaperone HSPA8. The polyubiquitinated p72 then interacts with the autophagy receptor SQSTM1/p62, leading to its degradation *via* the selective autophagy pathway. These findings reveal the mechanism of p72 degradation through autophagy and provide new insights into the capsid disassembly process of ASFV.

## INTRODUCTION

African swine fever (ASF) is a highly contagious disease affecting domestic pigs and wild boars, caused by ASF virus (ASFV) infection. It is characterized by high fever, skin cyanosis, and severe bleeding in the lymph nodes and internal organs. Acute infection with a virulent ASFV strain in domestic pigs results in 100% mortality (1). Recently, ASF has been distributed mainly in major economic zones with frequent trade in the pig industry (2). Various strains of ASFV have been isolated, including the highly virulent genotype II (by phylogenetic analysis of the viral p72 gene) (3, 4), lower virulence natural mutants (5), highly transmissible genotype I (6), and highly lethal genotype I and II recombinant ASFV strains (7). Unfortunately, no antiviral drugs or safe and effective commercial vaccines are currently available for the prevention and control of ASF.

ASFV is a complex nucleocytoplasmic large DNA virus characterized by an icosahedral multilayered structure, which is composed of a genome-containing nucleoid, a core-shell with thick protein, an inner lipid envelope, a capsid, and an external envelope, arranged from the innermost to outside (8). ASFV mainly infects monocytes, macrophages, and specific reticular cell lineages in the spleen, lymph nodes, lungs, kidneys, and liver (9). The major capsid protein p72, encoded by the ASFV *B646L* gene, constitutes approximately 31%–33% of the total mass of virions, making it one of the major antigens detected in infected pigs (8).

With the aid of pB602L, p72 is folded correctly and assembled to form a thermostable trimer (10). The inserts between the p72 folds of the trimer together form a propeller-like top structure that extends outside the virions, most likely the receptor-binding area for the cell surface (11). Previous study results showed that ASFV p72 on the surface of ASFV virions attaches to the cell membrane and is involved in the entry of ASFV (12). ASFV mainly uses two endocytic pathways to enter host cells: clathrin-mediated endocytosis and micropinocytosis (13, 14). Viral capsid proteins colocalize with EEA1 + early endosomes (EEs) from 1 to 30 min post-infection (mpi) and then translocate to CD63 + multivesicular bodies, RAB7 + late endosomes (LEs), and LAMP1 + lysosomes after 30 mpi. Most ASFV virions observed in late multivesicular bodies have shed the capsid, and this process requires a low intraluminal pH (15). From 45 mpi, the subvirions composed of naked cores can be detected in the cytoplasm and are released through pE248R and pE199L-mediated membrane fusion (14, 16, 17). ASFV DNA replication starts at 6 h post-infection (hpi) (15) and assembly occurs in perinuclear sites called viral factories (VFs), which are morphologically similar to cellular aggresomes (18).

Autophagy is a conserved “clearance” mechanism in eukaryotic cells that maintains cell homeostasis and orderly life activities through the degradation and recycling of intracellular components. Viral infections often induce autophagy to degrade the viral components or virions (19). Several autophagy receptors have been identified to be involved in this process (20). These receptors bind specifically to their substrates and induce their degradation *via* autophagy. Although the exact mechanism for selecting autophagy substrates is unknown, ubiquitination is considered a potential signal for misfolding and degradation of aggregators by autophagy. Sequestosome 1 (SQSTM1)/p62 is a ubiquitin (Ub)-targeted receptor that binds to the phagosome membrane *via* target-associated Ub and microtubule-associated protein 1 light chain 3 (MAP1LC3/LC3) (21). LC3 couples with lipid phosphatidylethanolamine (PE) to form the LC3-PE complex, completes the interchange between LC3-I and LC3-II, and participates in the formation of the autophagosome membrane, promoting its extension and closure to form mature autophagosomes (22). Eventually, the autophagosome fuses with a lysosome or LEs to degrade the target substrates. It has been reported that SQSTM1/p62 interacts with ubiquitinated tags and binds to the capsid of Chikungunya alphavirus, resulting in capsid degradation (23). However, the involvement of autophagy in the degradation of virions and viral proteins in ASFV remains unclear.

In this study, using anti-p72 antibodies- and NHS ester-labeled virions, we found ASFV virions were colocalized with autophagosomes and autolysosomes in porcine alveolar macrophages (PAMs) during the early phase of ASFV infection. Subsequently, we revealed that p72 degradation occurs *via* the autophagy pathway. E3 ligase Stub1 promotes p72 ubiquitination in an HSPA8-dependent manner. Polyubiquitinated p72 is recognized by the autophagy receptor SQSTM1/p62 to promote p72 degradation through selective autophagy. Notably, the autophagy-mediated degradation of p72 also contributes to capsid disassembly. Our findings revealed a novel mechanism of ASFV virion disassembly *via* autophagy during the early phase of ASFV infection.

## RESULTS

### Internalized ASFV virions enter autolysosomes during the early phase of viral infection

**Fig 1:**
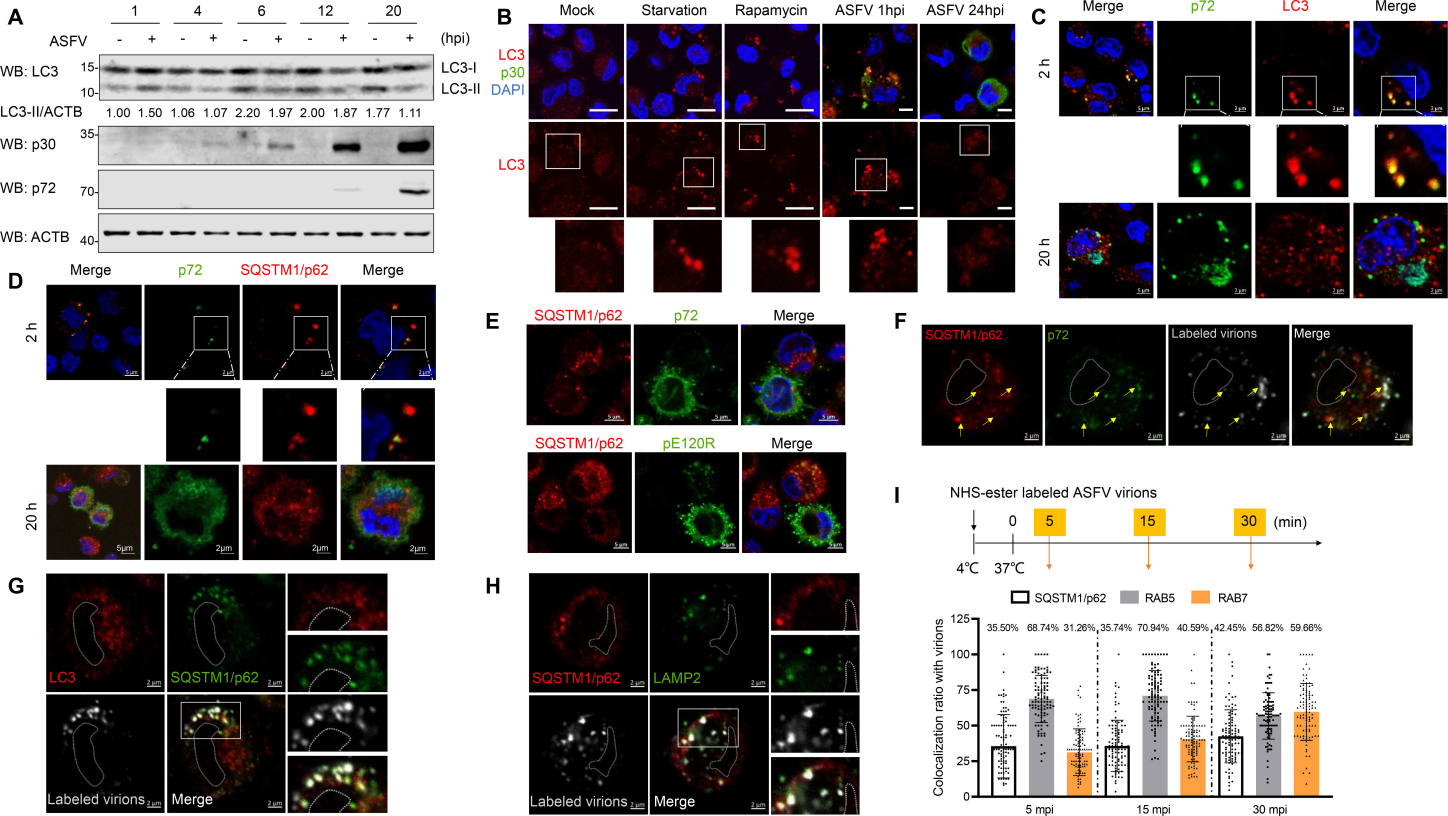
Internalized ASFV virions enter autolysosomes during the early phase of viral infection. (**A**) PAMs were infected with ASFV (multiplicity of infection [MOI] = 1) for the indicated time. The cells were lysed and the expressions of LC3, p72, and p30 were detected by western blotting. Relative protein levels were calculated using ImageJ software. (**B**) PAMs were starved in EBSS for starvation for 4 h or treated with 100 nM rapamycin for 24 h. The cells were mock-infected and infected with ASFV (MOI = 5, 1 h) or ASFV (MOI = 1, 24 h). The cells were stained with anti-LC3 and anti-p30 antibodies and observed by confocal microscopy. (**C**) PAMs were infected with ASFV (MOI = 5) for 2 h and ASFV (MOI = 1) for 20 h. The cells were stained with anti-p72 and anti-LC3 antibodies and then observed using a confocal microscope. (**D**) PAMs were infected with ASFV (MOI = 5) for 2 h or ASFV (MOI = 1) for 20 h, and immunofluorescence assay (IFA) was performed. The cells were stained with anti-p72 and anti-p62 antibodies and then observed using a confocal microscopy. (**E**) PAMs were infected with ASFV (MOI = 1) for 20 h and stained with the indicated antibodies. Colocalization of p72 and p62 or pE120R and p62 was observed by confocal microscopy. (**F–H**) PAMs were infected with Alexa Fluor 647 NHS ester-labeled ASFV (MOI = 10) (4°C, 1 h), washed, incubated (37°C, 30 min), and stained with anti-p72 and anti-p62 (**F**), anti-LC3 and anti-p62 (**G**), or anti-p62 and anti-LAMP2 (**H**) antibodies. The localizations were observed by confocal microscopy. (**I**) Infographic for experimental procedure (Top). PAMs were infected with Alexa Fluor 647 NHS ester-labeled ASFV (MOI = 10) (4°C, 1 h), washed, incubated (37°C, 5/15/30 min), and detected by IFA using anti-p62, anti-RAB5, and RAB7 antibodies, respectively (Bottom). The subcellular localization of ASFV virions and p62, RAB5, or RAB7 was observed by confocal microscopy. The percentage of virions colocalized with dot-like p62, RAB5, and RAB7 to virions, respectively, was calculated. In total, 100 cells were counted for each treatment. All results are presented as the mean ± SD form three independent experiments (ns, *P* > 0.05; *, *P* < 0.05; **, *P* < 0.01; ***, *P* < 0.001).

To investigate the effects of ASFV infection on the regulation of autophagy, the kinetics of the LC3 lipidation following ASFV infection was determined. PAMs infected with ASFV were harvested at 1, 4, 6, 12, and 20 h post-infection (hpi), respectively. We found that the expression of LC3-II was increased at 1 hpi, but subsequently decreased at 6, 12, and 20 hpi (Fig. 1A). LC3-II is present both inside and outside of autophagosomes (24). To enhance the comparative analysis of autophagosome formation in the early and late phases of ASFV infection, PAMs were infected with ASFV to examine the localization of LC3. As shown in Fig. 1B, in PAMs infected with ASFV for 1 h, the LC3 protein exhibited red fluorescent puncta that were comparable to those observed in the positive control group, which included starvation and rapamycin treatment, indicative of autophagosome formation. By contrast, no such puncta were detected in either uninfected cells or in PAMs infected with ASFV for 24 h. These findings suggest that ASFV infection induces immediate autophagosome formation in the early phase of infection, while inhibiting autophagy in the late phase of infection.

To assess whether the autophagosomes triggered by ASFV invasion (as an exogenous pathogen) co-localize with the virions or their components, PAMs were infected with 5 multiplicity of infection (MOI) of ASFV for 2 hpi and 1 MOI of ASFV for 20 hpi, and localization of p72 and LC3 was observed. As shown in Fig. 1C, anti-p72-labeled virions partially colocalized with the red fluorescent LC3 puncta at 2 hpi, but there was little colocalization of LC3 and newly synthesized p72 in the late phase of infection. SQSTM1/p62, like LC3, widely serves as hallmark markers for studying autophagy due to their critical roles in the autophagic process, their responsiveness to viral infections, and their ease of detection. Therefore, the localization of p72 and LC3 was observed, and we found that the autophagy receptor SQSTM1/p62 puncta colocalized with anti-p72-labeled virions at 2 hpi, but not at 20 hpi (Fig. 1D). To investigate the localization of viral particle proteins and autophagosomes in PAMs at different stages of ASFV infection under consistent treatment conditions, the anti-pE120R and anti-p72 antibodies were used to label the capsid proteins. Notably, it was observed that the capsid puncta co-localization with SQSTM1/p62 in cells at the early phase of infection, a pattern that was not evident in cells at the later phase of infection (Fig. 1E). These results indicate that the autophagosomes triggered by ASFV infection could co-localize with the virions in the early phase of infection. Previous studies have shown that purified virions can be covalently labeled with Alexa Fluor 647 NHS ester (25). To further demonstrate that it is the viral particles rather than the viral components that enter the autophagosome during the early phase of ASFV infection, PAMs were infected with NHS ester-labeled ASFV adsorbed at 4°C for 1 h, and then incubated at 37°C for 30 min, and the subcellular colocalization of ASFV virions, p72, and SQSTM1/p62 was detected. As shown in Fig. 1F, the co-localization of anti-p72 antibody-labeled spots, SQSTM1/p62, and NHS ester-labeled ASFV virions was observed. LC3 recruitment by SQSTM1/p62 is critical for the autophagosome formation. We tested whether the internalized ASFV virions colocalized with LC3 and SQSTM1/p62, and found that the colocalization of labeled virions, LC3, and SQSTM1/p62, could be observed (Fig. 1G). These results indicate that internalized ASFV virions can be transferred to autophagosomes during ASFV infection. Autophagosome-lysosome fusion is an essential step in cargo degradation (26). To further confirm whether ASFV virions entered autolysosomes, PAMs were infected with NHS ester-labeled ASFV virions for 30 min and the colocalization of virions, SQSTM1/p62, and LAMP2 (a lysosome marker) was detected. As shown in Fig. 1H, ASFV particles colocalized with SQSTM1/p62 and LAMP2, indicating that ASFV virions entered the autolysosomes during the early phase of infection.

After internalization, ASFV virions move along the endosome-lysosome pathway and are mainly found in the LEs and lysosomes of swine macrophages at 30 mpi, where ASFV particles undergo uncoating (14). To measure the proportion of internalized virions in SQSTM1/p62 + vesicles in ASFV-infected PAMs in the early phase of viral infection, the number of NHS ester-labeled ASFV in autophagosomes (labeled with SQSTM1/p62) and in endosomes (including RAB5 +EEs and RAB7 +LEs) was counted at 5, 15, and 30 mpi. As shown in Fig. 1I, approximately 70% of the NHS ester-labeled ASFV virions colocalized with RAB5-positive vesicles at 5 and 15 mpi, although the percentage was reduced at 30 mpi. Conversely, we observed that approximately 31.26%, 40.59%, and 59.66% of NHS ester-labeled ASFV virions colocalized with RAB7-labeled LEs at 5, 15, and 30 mpi, respectively. Consistently, 35.50%, 35.74%, and 42.45% of the virions colocalized with autophagosomes at 5, 15, and 30 dpi, respectively. Overall, these findings clearly showed that partially internalized ASFV virions can be transported to autophagosomes or autolysosomes in PAMs during the early phase of ASFV infection.

### Capsid protein p72 is targeted and degraded by autophagy

**Fig 2:**
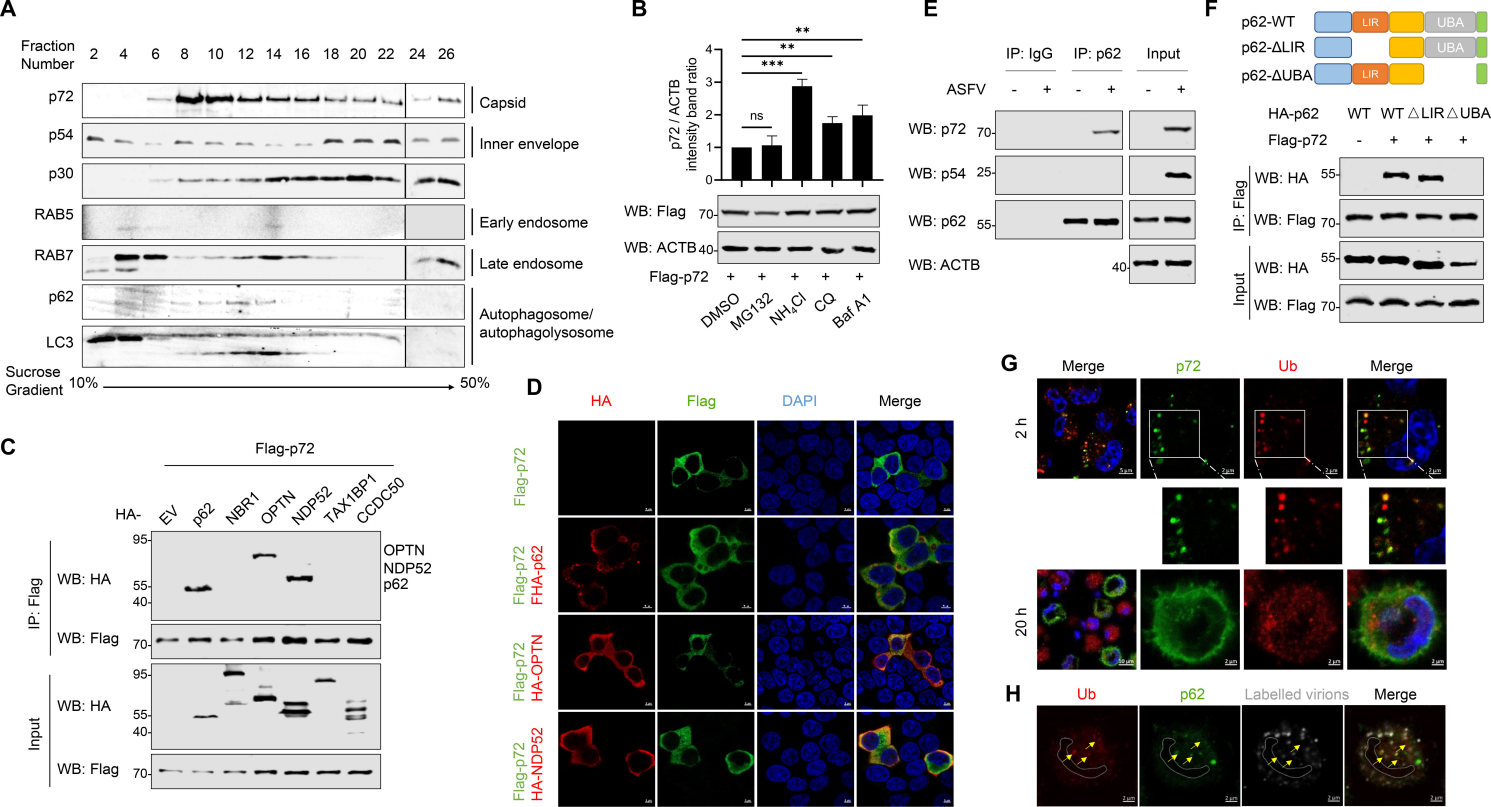
Capsid protein p72 is targeted and degraded by autophagy. (**A**) Sucrose density gradient separation of the lysates from PAMs infected with ASFV (MOI = 1) for 24 h. (**B**) HEK293T cells were transfected with a plasmid expressing FLAG-p72 and then treated with MG132 (10 µM), NH_4_Cl (10 mM), CQ (50 µM), and Baf A1 (100 nM), respectively. Thirty-six hours post-transfection (hpt), the cells were lysed and the expression of p72 was detected using western blotting. Relative amounts of protein were calculated using ImageJ software. (**C and D**) HEK293T cells were transfected with plasmids expressing FLAG-p72 and HA-tagged autophagy receptors, respectively. Co-IP was performed with an anti-FLAG antibody. Immunoprecipitates and whole-cell lysates (Input) were immunoblotted with anti-FLAG and anti-HA antibodies (**C**). The localizations of p72 and autophagy receptors were detected with immunofluorescence assay (IFA) using anti-FLAG and anti-HA antibodies by confocal microscope (**D**). (**E**) PAMs were mock-infected or infected with ASFV (MOI = 1) for 36 h, and then Co-IP was performed with an anti-p62 antibody. IgG was used as a negative control. (**F**) Schematic of p62 and its truncation mutants (Top). HEK293T cells were transfected with plasmids expressing p62 or its mutants, along with a plasmid expressing p72, and Co-IP was performed (Bottom). (**G**) PAMs were infected with ASFV (MOI = 5) for 2 h or ASFV (MOI = 1) for 20 h and IFA was performed. The cells were stained with anti-p72 and anti-Ub antibodies and then observed using a confocal microscopy. (**H**) PAMs were infected with Alexa Fluor 647 NHS ester-labeled ASFV (MOI = 10) (4°C, 1 h), washed, and incubated at 37°C for 30 min. The cells were stained with anti-Ub and anti-p62 antibodies and detected by IFA. The localization was observed by confocal microscopy. All results are presented as the mean ± SD from three independent experiments (ns, *P* > 0.05; *P < 0.05; **P < 0.01; ***P < 0.001).

To further demonstrate that ASFV virions can be localized to autophagosomes, we separated cell lysates from ASFV-infected PAMs using sucrose density gradient centrifugation. As shown in Fig. 2A, the capsid protein p72 and inner envelope protein p54 were found in the same fractions as the autophagy-related components (SQSTM1/p62, LC3, and RAB7) but not with RAB5. It has been reported that the disassembly and uncoating of ASFV virions depend on acidic pH (14), which can be achieved during the autophagy process. After capsid disassembly, ASFV virions undergo fusion to release their naked cores into the cytoplasm (14). To verify whether the main component of capsid proteins can be targeted by autophagy, HEK293T cells were transfected with a plasmid expressing FLAG-p72 and then treated with a proteasomal inhibitor (MG132) and lysosomal inhibitors such as Baf A1, chloroquine (CQ), and NH_4_Cl. As shown in Fig. 2B, all three lysosomal inhibitors inhibited p72 degradation, suggesting that p72 is degraded through the autophagy pathway. Autophagic cargo is selectively captured by autophagy receptors and transported to elongated autophagic membranes for selective degradation (20). SQSTM1/p62, NBR1, OPTN, NDP52, TAX1BP1, and CCDC50 have been identified as autophagy receptors (20). To determine which autophagy receptor controls autophagic degradation of p72, HEK293T cells were transfected with plasmids expressing the indicated proteins, and a co-immunoprecipitation (Co-IP) assay was performed. As shown in Fig. 2C, P72 mainly interacts with SQSTM1/p62, OPTN, and NDP52. We also found that only SQSTM1/p62 colocalized with p72 and formed aggregate in HEK293T cells (Fig. 2D; Fig S1A). The interaction between p72 and endogenous SQSTM1/p62 was confirmed in ASFV-infected PAMs (Fig. 2E), whereas p72 did not interact with endogenous OPTN and NDP52 (Fig S1B and C). The inner membrane protein p54, serving as a control, did not interact with any of these autophagy receptors. These results suggest that SQSTM1/p62 plays a significant role in p72-related autophagy.

Ubiquitinated substrates can be recognized by the Ub-binding domains (UBDs) present in autophagy receptors by binding to Ub. Previous studies have shown that the UBD (UBA) domain located at the C-terminus of SQSTM1/p62 recognizes ubiquitinated substrates (20). To determine which domain of SQSTM1/p62 is necessary for autophagic degradation of p72, two plasmids expressing SQSTM1/p62 mutants with the UBA domain deletion (ΔUBA) or LC3-interacting region (LIR) deletion (ΔLIR) were constructed. Co-IP results showed that the interaction between p72 and SQSTM1/p62 was significantly impaired when the UBA domain was lost, compared to SQSTM1/p62 (Fig. 2F). The UBA domain of SQSTM1/p62 is responsible for recognizing ubiquitinated substrates, which is also required for its interaction with p72. To test whether p72 is polyubiquitinated in ASFV-infected cells, PAMs were infected with ASFV for 2 or 20 hpi and stained with anti-p72 and anti-Ub antibodies. As shown in Fig. 2G, the colocalization results showed that the vast majority of Ub colocalized with p72 in dot form at 2 hpi, while few colocalized with newly synthesized p72 protein at 20 hpi. In addition, the colocalization of ASFV virions, SQSTM1/p62, and Ub was investigated in PAMs. As shown in Fig. 2H, the colocalization of NHS ester-labeled ASFV virions, SQSTM1/p62, and Ub was observed in ASFV-infected PAMs at 30 mpi, suggesting that p72 on the virions may be ubiquitinated and degraded by the ubiquitin system. Collectively, these findings demonstrate that p72 present in ASFV virions can be ubiquitinated and recognized by SQSTM1/p62 through its UBA domain in the early phase of viral infection.

### Stub1 affects p72 stability by promoting polyubiquitination of p72

**Fig 3:**
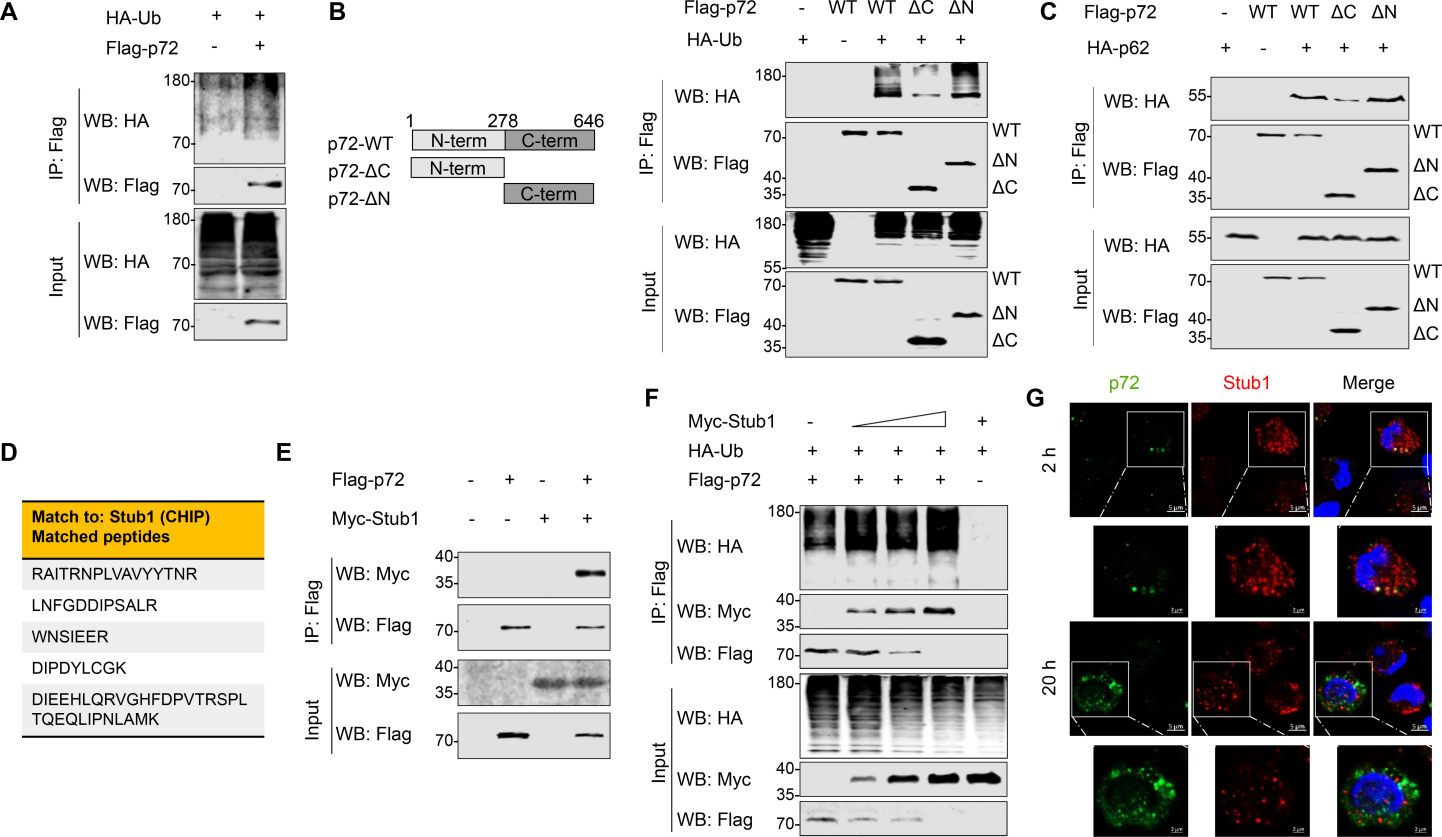
Stub1 affects p72 stability by promoting polyubiquitination of p72. (**A**) HEK293T cells were transfected with plasmids expressing FLAG-p72 and HA-Ub, and Co-IP analysis was performed to examine the interaction between p72 and Ub. (**B and C**) Schematic of p72 and its truncation mutants (B, left). Co-IP analysis of the interaction between FLAG-p72 or its truncation mutants and HA-Ub (B, right) or with HA-p62 (**C**) in HEK293T cells. (**D**) Identification of p72 binding partners by LC-MS/MS analysis. Peptides matched to the Stub1 amino acid sequence are listed in the table. (**E**) Co-IP analysis of the interaction between p72 and Stub1 in HEK293T cells. (**F**) HEK293T cells were transfected with plasmids FLAG-p72, HA-Ub, and increasing amounts of a plasmid expressing Myc-Stub1. At 36 hpt, Co-IP was performed to analyze the interaction between these proteins. (G) PAMs were infected with ASFV (MOI = 5) for 2 h or ASFV (MOI = 1) for 20 h. The cells were stained with anti-p72 and anti-Stub1 antibodies, and immunofluorescence assay (IFA) was performed.

To comprehensively elucidate the mechanisms by which p72 is targeted by autophagy, HEK293T cells were transfected with FLAG-p72 and HA-Ub plasmids, followed by Co-IP. We found that overexpressed p72 was polyubiquitinated in HEK293T cells (Fig. 3A). To determine which domain of p72 is necessary for its ubiquitination, two truncated mutants of p72 (p72-ΔC and p72-ΔN) were constructed. Domain mapping results showed that the C-terminal domain of p72 was primarily required for its ubiquitination (Fig. 3B). Indeed, the absence of the C-terminal domain of p72 significantly reduces the interaction with p62 (Fig. 3C). Taken together, our findings clearly demonstrate that SQSTM1/p62 recognizes polyubiquitinated p72, primarily at the C-terminus of p72.

To test which E3 ligase is required for p72 polyubiquitination, PAMs were infected with ASFV-HLJ/18, and Co-IP assays were performed using an anti-p72 antibody. Mass spectrometry (MS) results identified an E3 Ub ligase, Stub1, also named CHIP (12) (Fig. 3D). Co-IP results showed that Stub1 interacted with p72 in HEK293T cells (Fig. 3E). Furthermore, we investigated whether Stub1 was involved in p72 polyubiquitination and degradation. As shown in Fig. 3F, Stub1 overexpression significantly enhanced p72 polyubiquitination and reduced the protein level of p72 in HEK293T cells. Consistent with these results, we observed that endogenous Stub1 colocalized with p72 puncta at 2 hpi, but not with newly synthesized p72 that accumulated in VFs during the late phase of infection (Fig. 3G). In addition, we also investigated whether the expression of Stub1 is affected following ASFV infection. ASFV-infected PAMs were collected at 2, 6, 12, and 24 hpi, respectively, and the mRNA and protein levels of Stub1 were measured using quantitative PCR (qPCR) and western blotting. We found that the expression of Stub1 was not affected (Fig. S2). Collectively, these findings provide evidence that Stub1 interacts with p72 and promotes its ubiquitination and degradation of p72.

### Stub1 promotes the degradation of p72 through HSPA8-mediated autophagy

**Fig 4:**
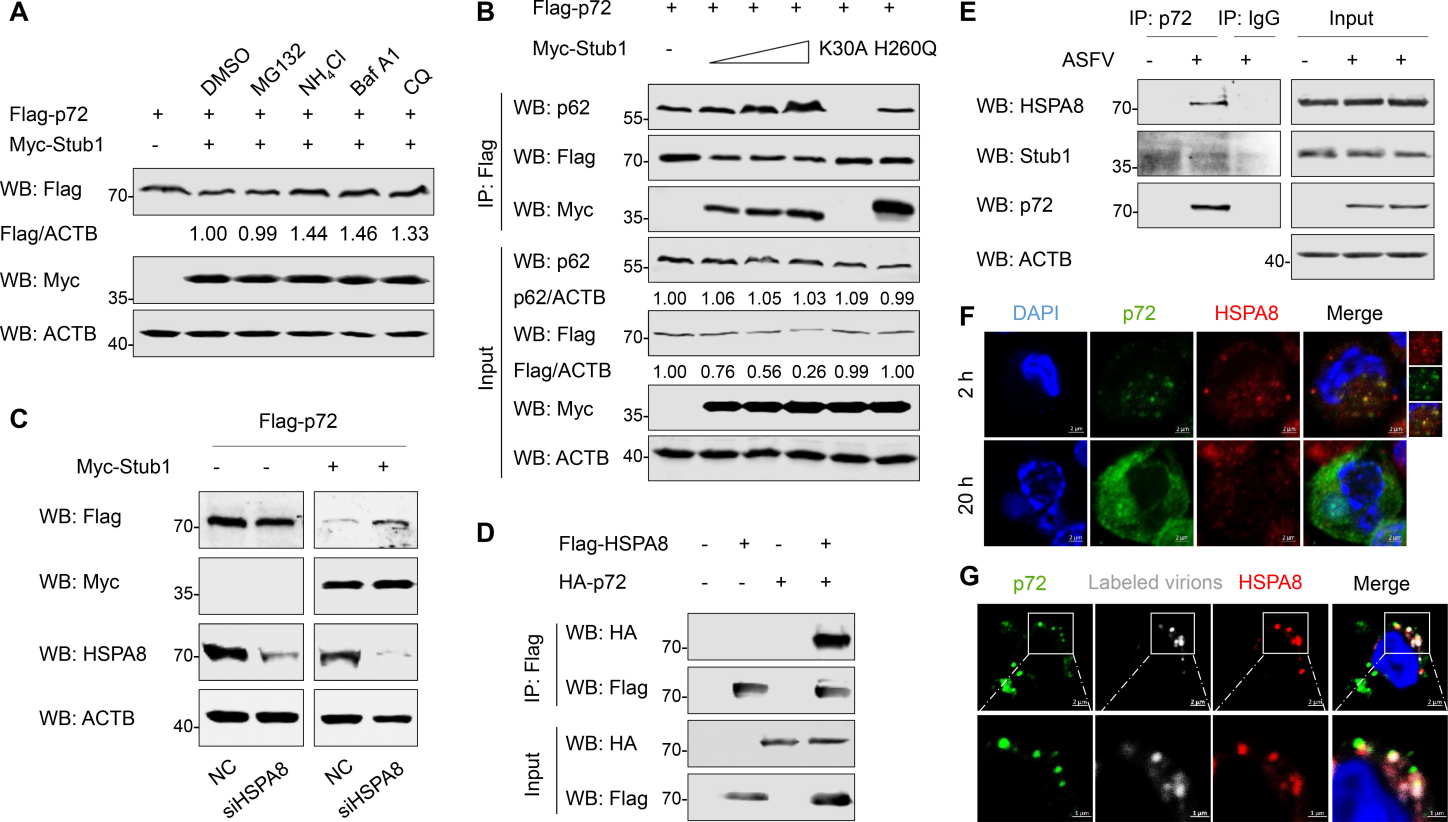
Stub1 promotes the degradation of p72 through HSPA8-mediated autophagy. (**A**) HEK293T cells were transfected with plasmids expressing FLAG-p72 and Myc-Stub1 and treated with MG132 (10 µM), NH_4_Cl (10 mM), Baf A1 (100 nM), and CQ (50 µM), respectively. At 36 hpt, the cells were lysed, and the expression of p72 was detected using the western blotting. Relative amounts of protein were calculated using ImageJ software. (**B**) HEK293T cells were transfected with a plasmid expressing FLAG-p72 and increasing amounts of a plasmid expressing Myc-Stub1 or plasmids encoding its mutants as indicated. At 36 hpt, Co-IP was performed to analyze the interaction between p62 and p72. Relative amounts of protein were calculated using ImageJ software. (**C**) HEK293T cells were transfected with plasmids expressing FLAG-p72 and Myc-Stub1 for 12 h, and then transfected with siRNAs targeting HSPA8 for another 36 h. p72 expression was detected using western blotting. (**D**) Co-IP analysis of the interaction between FLAG-HSPA8 and HA-p72 in HEK293T cells. (**E**) PAMs were mock-infected or infected with ASFV (MOI = 1) for 36 h, and then Co-IP was performed with an anti-p72 antibody. IgG was used as a negative control. (**F**) PAMs were infected with ASFV (MOI = 5) for 2 h and ASFV (MOI = 1) for 20 h. The cells were stained with anti-p72 and anti-Stub1 antibodies and observed using confocal microscopy. (**G**) PAMs were infected with Alexa Fluor 647 NHS ester-labeled ASFV (MOI = 10) (4°C, 1 h), washed, incubated at 37°C for 30 min, and then stained with anti-p72 and anti-HSPA8 antibodies. The localization was observed by confocal microscopy.

Previous studies have shown that E3 ligase Stub1 specifically targets ubiquitinated protein aggregates for autophagic clearance (27). To investigate how Stub1 affects p72 stability, HEK293T cells co-expressing FLAG-p72 and Myc-Stub1 were treated with proteasomal and autophagy-lysosomal inhibitors, respectively. As shown in Fig. 4A, Stub1-mediated p72 degradation was inhibited by autophagy-lysosomal inhibitors but not by proteasomal inhibitors. These results indicate that Stub1 mediates p72 degradation through autophagy.

The H260 residue within the U-box domain is crucial for E3 ligase activity of Stub1 (28). Stub1 with a point mutation in the TPR chaperone-binding domain (Stub1_K30A_) cannot bind to its chaperones, resulting in its substrate binding ability being significantly reduced (29). Subsequently, to investigate whether the E3 ligase and chaperones binding activity of Stub1 was required for the ubiquitination of p72, HKE293T cells were transfected with FLAG-p72 and Myc-Stub1 mutants, followed by Co-IP to assess the expression of p72 and its interaction with endogenous SQSTM1/p62. As shown in Fig. 4B, in contrast to Stub1-WT, Stub1_K30A_ and Stub1_H260Q_ did not promote p72 degradation. In addition, more endogenous SQSTM1/p62 interactions with p72 were detected with increasing Stub1-WT expression, whereas the interaction and p72 degradation were significantly weakened in cells expressing Stub1_K30A_ or Stub1_H260Q_. These results indicate that both H260 and K30 of Stub1 are essential for autophagy-mediated p72 degradation.

The heat shock protein family A member 8 (HSPA8) is a key chaperone involved in selective autophagy. Stub1 has been identified as a cofactor of HSPA8, which also participates in selective autophagy-mediated degradation of ubiquitinated substrates (30). To test whether HSPA8 is involved in Stub1-mediated p72 degradation, HEK293T cells were transfected with plasmids expressing FLAG-p72 and Myc-Stub1 for 12 h, and then transfected with small interfering RNA (siRNA) targeting HSPA8 (siHSPA8) for 36 h. The results showed that knockdown of HSPA8 expression inhibited Stub1-mediated degradation of p72 (Fig. 4C). In addition, there was an interaction between p72 and HSPA8 in HEK293T cells (Fig. 4D), which was confirmed in ASFV-infected PAMs (Fig. 4E). To test the colocalization of HSPA8 and p72, PAMs were infected with 5 MOI of ASFV for 2 h and 1 MOI of ASFV for 20 h, and their localization was observed by confocal analysis. Colocalization of HSPA8 and anti-p72-labeled virions was observed at 2 hpi, whereas HSPA8 did not show obvious colocalization with the diffusely distributed newly synthesized p72 at 20 hpi (Fig. 4F). Consistently, NHS ester-labeled ASFV particles colocalized with HSPA8 and p72 during the early phase of infection (Fig. 4G). These results suggested that HSPA8 interacts with p72 on the surface of ASFV virions. As a cofactor of HSPA8, Stub1 promotes ubiquitination and autophagy-mediated degradation of p72.

### p72 degradation by HSPA8-mediated autophagy contributes to capsid disassembly

**Fig 5:**
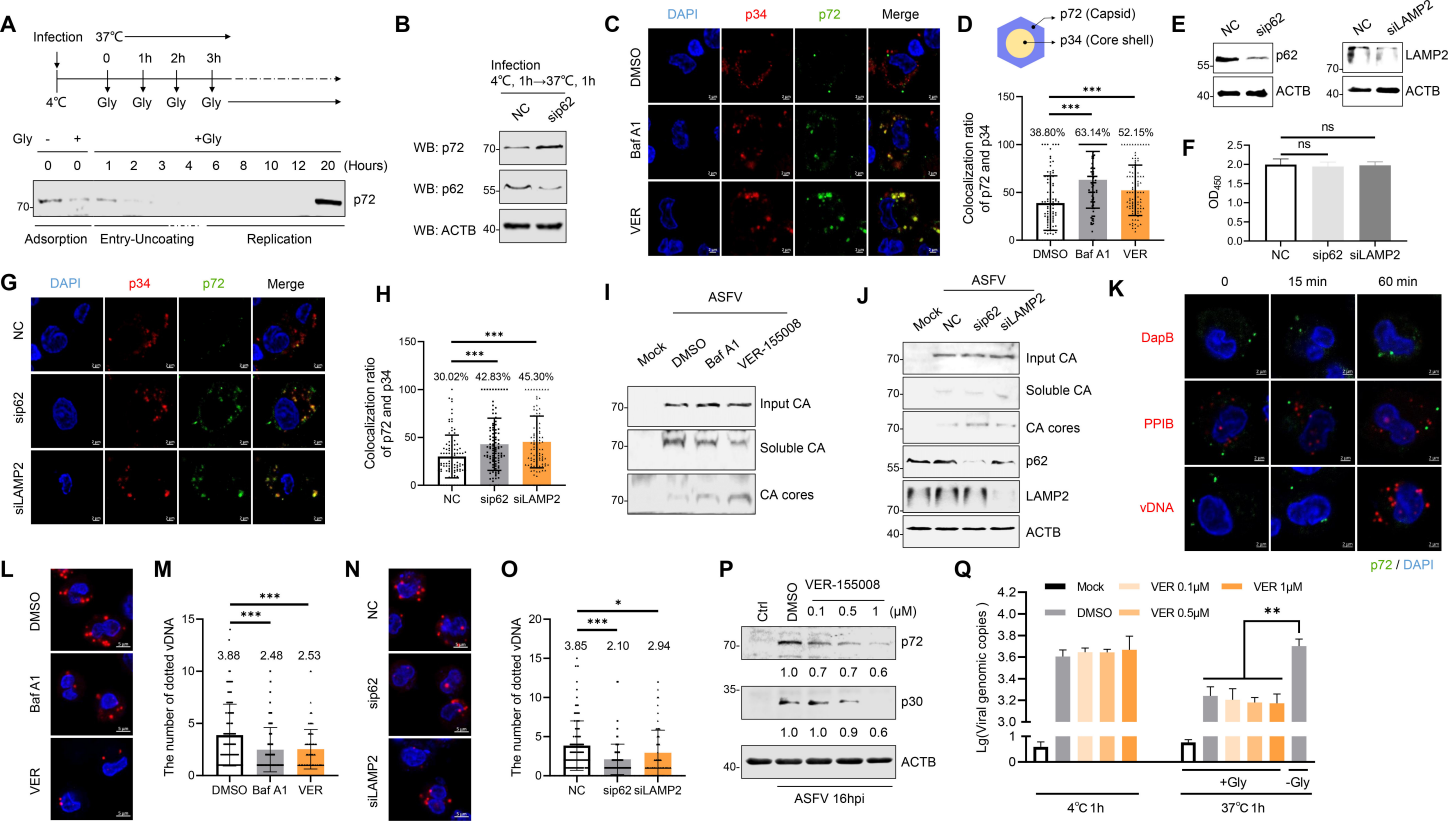
p72 degradation by HSPA8-mediated autophagy contributes to capsid disassembly. (**A**) Schematic of the experimental procedure (Top). PAMs were infected with ASFV (MOI = 10) (4°C, 1 h) and then incubated at 37°C for the indicated time. The cells were treated with 0.2 M glycine (pH 3.0) for 10 min at room temperature, washed, and lysed. The lysates were detected using western blotting (Bottom). (**B**) PAMs were transfected with siRNAs targeting p62 for 36 h, and then infected with ASFV (MOI = 10) (4°C, 1 h, followed by incubation at 37°C for 1 h. The cells were lysed, and the protein lysates were concentrated using TCA precipitation. The expression of p72 and p62 was detected using western blotting. (**C and D**) PAMs were pretreated with Baf A1 (100 nM) or VER-155008 (1 µM) for 12 h, and then infected with ASFV (MOI = 10) (4°C, 1 h), washed, incubated at 37°C for 30 min, and stained with anti-p72 or anti-p34 antibodies. The localization was observed by confocal microscopy (**C**). The proportion of p34 colocalized with p72 to p34 was calculated, and 100 cells were counted for each treatment (**D**). (E–H) PAMs were transfected with siRNAs targeting p62 or LAMP2 for 36 h, and then infected with ASFV (MOI = 10) (4°C, 1 h), washed, incubated at 37°C for 30 min, and stained with anti-p72 or anti-p34 antibodies (**G**). The expression of p62 and LAMP2 was detected using western blotting (**E**) and the cell viability was analyzed using a CCK-8 counting kit (**F**). The ratio of p34 colocalized with p72 to p34 was calculated, and 100 cells were counted for each treatment (**H**). (**I and J**) PAMs pretreated with Baf A1 (100 nM) or VER-155008 (1 µM) for 12 h (I), or transfected with siRNAs targeting p62 or LAMP2 for 36 h (J). The cells were infected with ASFV (MOI = 10) (4°C, 1 h), washed, and incubated at 37°C for 2 h, and the fate-of-capsid assays were performed. (**K**) PAMs were infected with ASFV (MOI = 10) (4°C, 1 h), washed, and incubated at 37°C for 0, 15, or 60 min. ASFV vDNA was detected using DNAscope ISH with V-ASFV-C962R-I215L-C1 probes and RNAscope Multiplex Fluorescent Reagent Kit. DapB was used as a negative control. Ss-PPIB was used as a positive control. The cells were stained with an anti-p72 antibody. (**L-O**) PAMs pretreated with Baf A1 (100 nM) or VER-155008 (1 µM) for 12 h (**L, M**), or transfected with siRNAs targeting p62 or LAMP2 for 36 h (**N, O**). The cells were infected with ASFV (MOI = 10) (4°C, 1 h), washed, incubated at 37°C for 60 min, and ASFV vDNA was detected using DNAscope ISH and was quantified. (**P**) PAMs were pretreated with DMSO or VER-155008 (0.1, 0.5, 1 µM) for 1 h and then infected with ASFV (MOI = 1) along with VER-155008 treatment for 16 h. The expression of p72 and p30 was detected using western blotting, and the relative amounts of proteins were calculated using ImageJ software. (**Q**) Virus attachment and internalization assays were performed in PAMs pretreated with DMSO or VER-155008 and the cells were then mock-infected or infected with ASFV (MOI = 5). The viral genomic copies were detected using qPCR. All results are presented as the mean ± SD from three independent experiments (ns, *P* > 0.05; *P < 0.05; **P < 0.01; ***P < 0.001).

Most ASFV virions lose their capsids in porcine macrophage multivesicular endosomes during ASFV uncoating (14). To study the initial steps leading to viral capsid disassembly during ASFV infection, we first detected changes in the p72 protein levels of the adsorbed or internalized virions. PAMs were infected with 10 MOI of ASFV, and p72 protein levels were monitored at various time points, as indicated by western blotting. The p72 level in the absorbed virions was detected by inoculation at 4°C, while the p72 level in the internalized viruses was detected after removing the absorbed viruses using glycine treatment (31), which allowed analysis of the kinetics of capsid protein levels during viral infection. As shown in Fig. 5A, P72 was degraded within the first 4 h of ASFV infection and was barely detectable after 4 h. At 20 hpi, newly synthesized p72 was detected because of viral genome amplification. To verify whether SQSTM1/p62 is involved in the degradation of p72 in the early phase of ASFV infection, PAMs were transfected with siRNA targeting SQSTM1/p62 (sip62) and then infected with ASFV for 1 h. The cells were lysed, and the protein lysates were concentrated using TCA precipitation for the detection of p72 expression (32). As shown in Fig. 5B, the endogenous expression of SQSTM1/p62 was significantly reduced and p72 expression was higher in cells transfected with sip62 compared to those transfected with siNC, suggesting that SQSTM1/p62 could be involved in the uncoating of ASFV in the early phase infection. Meanwhile, the siRNA-targeting Stub1 (siStub1) was synthesized, resulting in a significant reduction of endogenous Stub1 expression, with no adverse effects on cell viability observed following transfection with siStub1 (Fig S3A and B). To investigate whether the knockdown of Stub1 expression affects the degradation of p72, PAMs were transfected with siStub1 and subsequently infected with ASFV for 1 h. As shown in Fig S3C, Stub1 expression was significantly diminished, while p72 expression was elevated in cells transfected with siStub1 compared to those transfected with siNC. These findings suggest that the knockdown of Stub1 expression inhibits the degradation of p72 during the early phase of ASFV infection.

To further verify whether autophagy is involved in capsid disassembly during ASFV infection, PAMs were pretreated with VER-155008 (an HSPA8 inhibitor) and Baf A1 as a positive control and then infected with ASFV (MOI = 10). Colocalization of p72 and p34 (core proteins) was observed by confocal analysis. As shown in Fig. 5C and D, similar to Baf A1, VER-155008 incubation inhibited core-shell detachment from the capsid compared with control cells. To test whether SQSTM1/p62 and LAMP2 affect the disassembly of ASFV virions, PAMs were transfected with sip62 or siRNA targeting LAMP2 (siLAMP2), and then infected with ASFV. SQSTM1/p62 and LAMP2 protein expression was detected by Western blotting. We found that the endogenous expression of SQSTM1/p62 and LAMP2 was significantly reduced (Fig. 5E), and cell viability was not significantly affected (Fig. 5F) following transfection with siRNAs targeting SQSTM1/p62 and LAMP2. As shown in Fig. 5G and H, the knockdown of SQSTM1/p62 or LAMP2 expression increased the proportion of p72 and p34 colocalization in ASFV-infected PAMs. These data suggest that autophagy is involved in capsid disassembly. Furthermore, PAMs were infected with ASFV (MOI = 10) and incubated with VER-155008, and the stability of viral capsid (CA) cores was assessed using a fate-of-capsid assay (33). Soluble CA, which is indicative of fully uncoated and intact CA cores, was separated using ultrafast centrifugation. Compared to the control group, the expression of soluble CA decreased in PAMs pretreated with VER-155008 and Baf A1, whereas the expression of CA cores increased (Fig. 5I). Similar results were obtained when PAMs were transfected with sip62 and siLAMP2 and then infected with ASFV (MOI = 10) (Fig. 5J).

Next, we used highly specific *in situ* hybridization (ISH) with single-copy sensitivity (DNAscope) to detect and quantify viral DNA (vDNA) which was released after capsid disassembly of internalized ASFV in PAMs. PAMs were infected with ASFV (MOI = 10) for 0, 15, 60 min. We found that there was no detectable vDNA release within 15 min of infection, but vDNA was detected at 60 mpi (Fig. 5K). As a positive control, SsPPIB was detected at each time point, while DapB was not detected as a negative control. Inhibition of p72 degradation through autophagy affected virus uncoating, and it is speculated that it may also inhibit subsequent viral genome release. To test whether the released vDNA of ASFV is decreased in the presence of autophagy inhibitors, PAMs were pretreated with VER-155008 and Baf A1 and infected with ASFV for 60 min. We found that the released vDNA of ASFV was decreased in the presence of the indicated inhibitors for autophagy (Fig. 5L and M). Similar results were obtained when PAMs were transfected with sip62 and siLAMP2 and then infected with ASFV (Fig. 5N and O). These results indicate that blocking autophagy inhibits ASFV genome release. To further examine the effects of HSPA8 inhibition on ASFV replication, PAMs were pretreated with VER-155008 and infected with ASFV for 16 h. We found that the expression levels of viral protein p72 were reduced in a dose-dependent manner at 16 hpi in cells pretreated with VER-155008 (Fig. 5P). In addition, ASFV attachment and internalization were not affected (Fig. 5Q).

**Fig 6:**
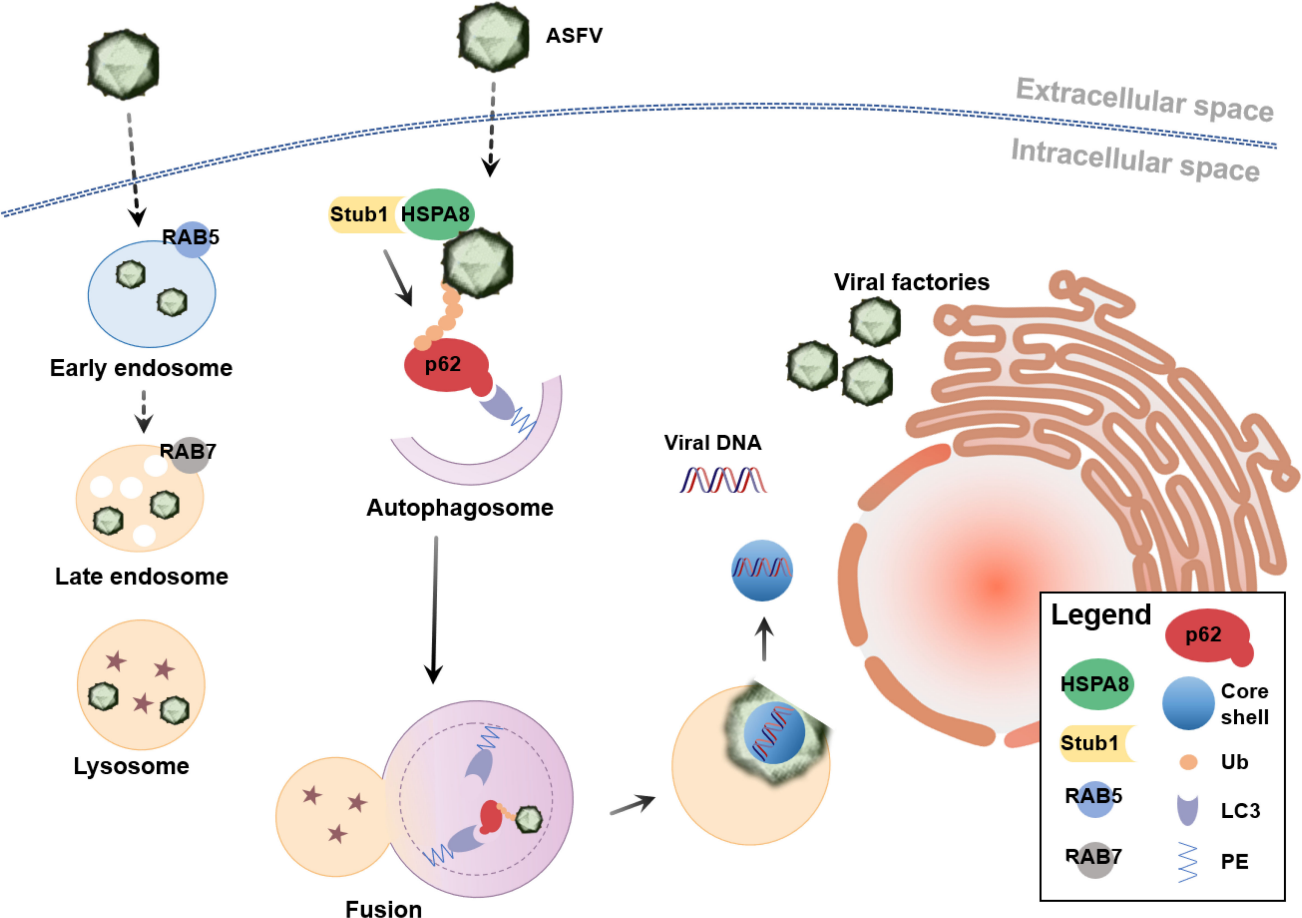
Model of the role of autophagy in ASFV capsid disassembly. After ASFV virions enter cells, internalized virions are transported from early endosomes to late endosomes. Stub1 promotes the ubiquitination of p72 on the surface of virions; subsequently, ubiquitinated p72 can be recognized by the autophagy receptor p62 in an HSPA8-dependent manner. Virions are captured and directed to autophagosomes, which fuse with late endosomes/lysosomes to degrade p72. Capsid disassembly is also observed. The inner envelope proteins fused to the endosomes/lysosomes membrane deliver genome-containing cores into the cytosol.

Taken together, we propose that interference with critical autophagic adaptors affects p72 degradation by regulating the capsid disassembly process, resulting in the inhibition of ASFV replication in the early phase of infection (Fig. 6).

## DISCUSSION

The ASFV infection cycle begins with viral adsorption and its entry into host cells. Once the virus enters the endocytic pathway, ASFV particles become uncoated (34, 35) within multivesicular endosomes, leading to the loss of the outer lipid envelope and protein capsid (36). The major capsid protein, p72, in ASFV virions is the most dominant structural component (8). However, mechanisms underlying ASFV disassembly and p72 degradation remain unclear. In this study, we found that internalized ASFV virions entered autolysosomes during the early phase of viral infection. We found that p72 is ubiquitinated and captured by the autophagy receptor p62. The E3 ubiquitin ligase Stub1 promotes the ubiquitination and degradation of p72 mediated by HSPA8-dependent selective autophagy.

Autophagy ensures the degradation of cytosolic substrates *via* the lysosomal pathway. During viral infection, autophagy acts as a surveillance and cell-intrinsic defense mechanism that delivers virions or viral constituents to endosomal/lysosomal compartments enriched in immune sensors (20, 37). For example, the capsid protein VP2 of the foot-and-mouth disease virus induces autophagy through the EIF2S1-ATF4-AKT-MTOR cascade (38). Similarly, the capsid protein VP2 in avibrinaviruses is degraded *via* selective autophagy (39). ASFV structural proteins such as pE199L, pK205R, and p17 have been reported to be associated with the induction of autophagy, while their degradation by autophagy has not been studied (40–42). Recently, FoxJ1 degrades ASFV pMGF505-2R and pE165R through the autophagy pathway and positively regulates the innate immune response to play an antiviral role against ASFV replication (43). Sorting nexin 32 (SNX32) degrades p30 by recruiting the autophagy-related protein Ras-related protein Rab-1b (RAB1B) to inhibit ASFV growth and replication (44). Although ASFV encodes more than 150 proteins, there are few studies on the degradation of ASFV components by autophagy. In this study, autophagosome formation was primarily observed in PAMs during the early phase of ASFV infection (Fig. 1). We also observed that ASFV virions entered autolysosomes, suggesting that autophagy may be stimulated by structural proteins in ASFV virions. We noticed that p72 interacts with SQSTM1/p62, and LC3-labeled autophagosomes colocalized with dot-like p72 in ASFV-infected PAMs, especially in the early phase of infection. Interestingly, p72 was modified by polyubiquitin and promoted its degradation by Stub1, which can be inhibited in the presence of autophagolysosome inhibitors. This suggests that autophagy targets p72, causing the engulfment of ASFV virions by autophagosomes.

Many autophagy substrates specifically and exclusively target autophagosomes *via* autophagy receptors, which is considered selective autophagy. Autophagy receptors attach ubiquitinated cargo to nascent autophagosome-carried LC3, resulting in protein degradation *via* selective autophagy (27). The autophagy receptor p62 plays a vital role in selective autophagy by targeting ubiquitinated proteins or pathogens to phagophores for degradation (27). Previous studies have demonstrated that p62 interacts with and promotes the degradation of other viral constituents such as VP1 and VP3 of Seneca Valley virus (SVV) (45), capsid protein VP2 of avibrinavirus (39), and capsid protein of Dengue virus (46). In this study, SQSTM1/p62 was identified as the primary autophagy receptor responsible for mediating the degradation of p72 *via* its UBA domain.

Stub1/CHIP (carboxyl terminus of the HSPA8-interacting protein) belongs to the U-box family of E3 Ub ligases (47). Some host and viral proteins are ubiquitinated by Stub1 and subsequently degraded through the proteasome or autophagy pathways. For example, the human immunodeficiency virus-1 (HIV-1) accessory protein VIF interacts with Stub1, leading to its ubiquitination and degradation (48). Remarkably, Stub1 also functions as a chaperone-dependent E3 ligase (47) and promotes the ubiquitination and degradation of the host proteins TAp63 (49), ASK1 (50), and AGO2 (51) through its E3 ligase and chaperone-binding activities. These degradation processes occur *via* the proteasomal system, the first two of which are dependent on the chaperone heat shock protein 70 (HSP70). Oxidatively stressed peroxisomes (52) and HIF1A (53) of the host and the induced aberrant polymers of Hepatitis B virus capsid proteins (54) are removed by Stub1 *via* the autophagy pathway, which is mediated by the chaperone HSPA8 (HSC70). In this study, Stub1 was identified as one of the p72 binding partners by IP-MS, and Stub1 was found to promote p72 ubiquitination and degradation *via* p62-mediated autophagy *via* HSPA8.

Members of the HSP70 family of chaperones are involved in all phases of the viral life cycle in viruses such as enterovirus and dengue virus (55, 56). HSPA8 is an abundant cytosolic isoform of HSP70 that reverses protein aggregation and disassembles protein complexes (57). HSPA8 is involved in particle disassembly in several viruses, such as reovirus (58) and cucumber necrosis virus (CNV) (59). Previous studies have shown that HSPA8 is a component of ASFV particles, detected using LC-MS/MS (8, 60), which may be incorporated into virions or pulled down as virion-binding proteins. In this study, we revealed that p72 polyubiquitination by Stub1 was recognized by p62, which mediated p72 degradation by autophagy *via* HSPA8 during the early phase of ASFV infection.

An acidic pH may also provide chemical cues for viral uncoating, triggering ASFV disassembly (61). The fusion of autophagosomes with lysosomes or late endosomes creates an acidic environment that can be exploited by a virus for capsid disassembly. In this study, we observed that 35%–45% of ASFV virions could be localized in p62-labeled autophagosomes within 30 mpi, suggesting that the degradation of p72 through autophagy may be related to viral capsid disassembly. Currently, the technical methods for detecting viral decapsidation are limited. ASFV capsid p72 and core p150 are localized in different layers of virions. Intact virions were only stained with anti-p72 and anti-p150 antibodies, whereas decapsidated virions were only stained with the anti-p150 antibody. Based on this information, a decapsidation assay was conducted to calculate the ratio of decapsidated to intact ASFV virions (62). In addition, a fate-of-capsid assay was established to separate soluble capsids, indicative of fully uncoated and intact capsid cores, through ultrafast centrifugation, which has been used in research on HIV-1 uncoating (33). Treatment of PAMs with HSPA8 inhibitors and/or siRNAs targeting autophagy-associated proteins, in combination with the methods described above, demonstrated that p72 degradation through HSPA8-mediated autophagy was related to capsid disassembly. The inhibition of this process clearly affects the release of viral genomic DNA and the expression of early viral proteins. Previous studies have shown that internalized ASFV virions enter the endosome and are then transferred through the endosome-lysosome pathway (14, 15, 61). Moreover, ASFV infection inhibits autophagy (1, 63), except during the early phase of infection. Based on these results, we propose a model and show that autophagy provides favorable conditions for the virus to undergo uncoating and release viral genomes. The endosomal-dependent “normal procedure” is not the only mechanism of ASFV capsid degradation, and viruses may compensate for capsid removal through other mechanisms.

In summary, our findings revealed that we found that internalized ASFV virions entered autolysosomes during the early phase of viral infection. Stub1 promotes p72 ubiquitination and degradation through the HSPA8-mediated autophagy pathway, providing insights into the mechanism of p72 degradation and offering a model for understanding the involvement of autophagy in capsid disassembly during ASFV infection.

## MATERIALS AND METHODS

### Cells and viruses

PAMs isolated from the lung lavage fluid of 4-week-old specific pathogen-free piglets were cultured in RPMI-1640 medium containing 10% heat-inactivated fetal bovine serum (FBS; Hyclone), 100 U/mL penicillin, and 50 mg/mL streptomycin (Gibco). HEK293T cells were purchased from the American Type Culture Collection and cultured in Dulbecco’s Modified Eagle’s medium supplemented with 10% heat-inactivated FBS. All the cells were maintained at 37°C with 5% CO_2_. ASFV strain HLJ/18 (GenBank accession number MK333180.1) was amplified from porcine bone marrow cells.

### Plasmids, antibodies, and reagents

Plasmids expressing FLAG-p72, HA-p72, HA-Ub, and FLAG-HSPA8 were constructed and stored in the laboratory. Complementary DNAs (cDNAs) encoding host proteins and their mutants were obtained by reverse transcription-PCR (RT-PCR) from total RNA extracted from PAMs. The cDNAs corresponding to p62, p62-ΔLIR, p62-ΔUBA, Stub1, Stub1-K30A, Stub1-H260R, NBR1, OPTN/ NDP52, TAX1BP1, and CCDC50 were amplified by PCR and cloned into pCAGGS vectors with different tags (FLAG, HA, and Myc). Viral genomic DNA was extracted from ASFV-infected PAMs using a DNA Mini Kit (Qiagen). The cDNAs corresponding to p72-ΔC, and p72-ΔN, were amplified from the viral DNA and cloned into pCAGGS vectors with different tags (FLAG, HA, and Myc). Mouse anti-p72, p30, and pE120R polyclonal antibodies (pAbs) and rabbit anti-p54 and p34 pAbs were prepared from mice or rabbits immunized with recombinant proteins. Other commercially available antibodies are shown in Table S2, and the dilution ratio of the antibodies in the western blotting and immunofluorescence assay (IFA) test refers to product usage information. Goat anti-mouse and anti-rabbit IgG antibodies (Dylight 800) were purchased from CST. Goat anti-mouse and anti-rabbit IgG with highly cross-adsorbed secondary antibodies (Alexa Fluor 594/488) were purchased from Thermo Fisher Scientific. Chloroquine, Bafilomycin A1, MG132, and Rapamycin were purchased from MedChemExpress. Earle’s Balanced Salt Solution was purchased from Gibco.

### Co-IP and western blotting

PAMs were infected with ASFV or pretreated with the indicated inhibitors or HEK293T cells were transfected with plasmids. Cells were harvested and lysed in 1% Nonidet P-40 buffer (50 mM Tris pH 7.4, 1 mM EDTA, 12.5 mM NaCl, 1% Nonidet P-40, 6 mM Mg2Cl, and 10% glycerol) containing a protease inhibitor cocktail. The lysates were collected for western blotting and Co-IP. Cell lysates from PAMs were immunoprecipitated using the indicated specific antibodies and incubated with protein A/G Sepharose beads. Lysates from HEK293T cells transfected with FLAG-tagged plasmids were immunoprecipitated using anti-FLAG (M2) agarose beads. Protein extracts were separated by sodium dodecyl sulfate–polyacrylamide gel electrophoresis and electrotransferred to polyvinylidene fluoride membranes (GE Healthcare), which were blocked in Tris-buffered saline containing 5% skim milk and incubated with the indicated antibodies. Secondary antibodies were conjugated to DyLight 800, and immunoblotting was performed using IRDye 680LT (LI-COR).

### Immunofluorescence assay

PAMs were infected with ASFV or pretreated with the indicated inhibitors or HEK293T cells were transfected with plasmids. The cells were then washed with phosphate-buffered saline (PBS), fixed with 4% paraformaldehyde, and permeabilized with 0.3% Triton X-100. After blocking with 10% FBS, immunolabeling was performed using primary antibodies, followed by secondary antibodies. Nuclei were stained with DAPI (Solarbio). Immunofluorescence images were acquired using a laser-scanning confocal microscope (Leica SP2).

### Sucrose density gradient centrifugation assay

Cells were lysed in homogenization buffer (50 mM Tris-HCl [pH 7.4], 250 mM sucrose, 25 mM KCl, 5 mM MgCl2, 3 mM imidazole) supplemented with protease inhibitor cocktail and phosphatase inhibitor on a rocking platform at 4° for 30 min. Subsequently, cells were scraped and centrifuged at 12,000 × *g* for 10 min. The pellet was discarded, and the supernatant was loaded on a continuous sucrose gradient (10%–50% sucrose) and centrifuged 194,000 × *g* for 16 h. Each sample was divided into 26 fractions and every second fraction was analyzed by western blotting.

### Mass spectrometry

The ASFV p72-binding proteins in the immunoprecipitants were resolved by SDS-PAGE and silver staining. The specific bands were cut and processed for liquid chromatography-tandem mass spectrometry (LC-MS/MS) (probability-based protein identification by searching sequence databases using mass spectrometry data). The MS/MS signals were then processed against the National Center for Biotechnology Information (NCBI) protein database using the Mascot Server (Matrix Science). High-confidence peptides with a prerequisite of a minimum of two peptides leading to the identification of proteins were selected and listed in Table S3. The protein “score” displays the standard score, the cumulative protein score based on summing the ion scores of the unique peptides. A higher score indicates higher confidence in identification.

### Quantitative PCR

Total RNA was extracted using TRIzol reagent (Invitrogen) and reverse transcript to cDNA by reverse transcriptase (Takara). cDNAs were prepared for the RT-PCR using SYBR Green Premix detection system. On the side, for ASFV genomic copy detection, viral genomic DNA was extracted from ASFV-infected PAMs using a DNA Mini Kit (Qiagen). RT-PCR using TaqMan (Takara) was performed on a QuantStudio5 system (Applied Biosystems) according to the procedure recommended by King et al. (64). The primers used are listed in Table S1.

### RNA interference

The targeting sequences of the siRNAs used in this study were synthesized by GenePharma and are listed in Table S1. The cells were transfected with different siRNAs using Lipofectamine RNAiMAX Transfection Reagent (Thermo Fisher Scientific) according to the manufacturer’s instructions. PAMs were transfected with siRNAs for 36 h and then infected with ASFV. The efficiency of the siRNA knockdown of the target protein was assessed using qPCR or western blotting.

### ASFV virion purification and labeling

Infect suitable cells with ASFV and allow the virus to propagate. Harvest the supernatant containing the virus after a suitable incubation period (typically 72 h). Centrifuge the cell culture supernatant at low speed (e.g., 3,000 rpm for 10–15 min) to remove cell debris and other large particles. Collect the supernatant and centrifuge at 38,000 × *g* for 1.5 h at 4°C. Suspend the viral pellet with PBS. Prepare a sucrose gradient ranging from 33% at the top to 56% at the bottom in an ultracentrifuge tube. Carefully load the clarified supernatant onto the top of the sucrose gradient without disturbing the layers. Centrifuge the gradient at high speed (e.g., 80,000 *× g*) for several hours (typically 1–3 h) at 4°C. After centrifugation, carefully collect fractions from the gradient using a pipette or a fraction collector. The virus will typically band at a specific density, often around 40%–50% sucrose, but this can vary. Analyze the collected fractions for the presence of ASFV by HAD_50_ to determine virus titers.

The purified ASFV virions were labeled with Alexa Fluor 647 NHS ester (ThermoFisher) (100 µM) (20°C, 1 h) in the dark. The labeling system was vortexed every 15 min, unincorporated dye was removed using an Amicon Ultra-15 centrifugal filter unit (30 kDa) (Merck Millipore) by centrifugation at 6,000 × *g* for 30 min at 4°C, and a wash step repeated three times using PBS. Finally, NHS ester-labeled ASFV virions were filtered through a 0.45 µm pore filter (Millipore). The labeled purified ASFV was aliquoted and stored at −80°C.

### Viral attachment and internalization assay

PAMs were pretreated with DMSO or VER-155008 (0.1, 0.5, and 1 µM) for 12 h and then infected with ASFV (MOI = 5) and incubated at 4°C for 1 h. Unbound ASFV virions were removed by washing three times with PBS. Samples for the attachment assay were collected, and viral genomic DNA was extracted for qPCR. The cells for the internalization assay were incubated in the medium with VER-155008 at 37°C for 1 h and treated with 0.2 M Glycine (pH 3.0) for 10 min at room temperature. Then the cells were washed three times to remove extracellular viruses. Samples were collected and the viral genomic DNA was extracted for qPCR.

### Fate-of-capsid assay

PAMs were pretreated with DMSO, Baf A1, or VER-155008 for 16 h, and then infected with ASFV (MOI = 10) at 4°C for 1 h to allow adequate attachment and then at 37°C for 2 h with the inhibitors. The cells were lysed using hypotonic lysis buffer (20 mM HEPES pH 7.9, 10 mM KCl, 0.2% Nonidet P-40, 10% glycerol) containing protease inhibitors and a Dounce homogenizer, and briefly centrifuged to clarify the cytosolic fractions. The lysates were then layered on a 50% sucrose cushion and ultracentrifuged at 100,000 × *g* for 2 h. Soluble CA (top fraction) and intact cores (pellet) were analyzed by western blotting using an anti-p72 pAb. For RNA interference, PAMs were transfected with siRNAs for 36 h and then infected with ASFV (MOI = 10), and the above procedure was repeated.

### ASFV vDNA detection using DNAscope ISH

ASFV DNA in PAMs was detected *in situ* using RNAscope Probe-V-ASFV-C962R-I215L-C1 (Cat# 1311321-C1, Advanced Cell Diagnostics) and RNAscope Multiplex Fluorescent Reagent Kit (Cat# 323100, Advanced Cell Diagnostics) for color development. The RNAscope negative control probe-DapB (Cat# 310043, Advanced Cell Diagnostics) was used as a negative control. The RNAscope positive control probe-Ss-PPIB (Cat# 428591, Advanced Cell Diagnostics) was used as a positive control.

### Statistical analysis

Data are presented as mean ± SD. Statistical significance was analyzed using Student’s t-test. Statistical significance was set at *P* < 0.05. Significance values were set as follows: ns (not significant); *P* > 0.05; *P < 0.05; **P < 0.01; ***P < 0.001.

## Data Availability

The data supporting the findings of this study are available within the article and its Supplemental Material. In addition, the raw data and relevant analysis code are stored in a secure institutional repository at Harbin Veterinary Research Institute. Access to the data can be requested by contacting the corresponding author at wengchangjiang@caas.cn. The data will be provided upon reasonable request and subject to any necessary ethical and legal approvals. We have taken great care to ensure the integrity and security of the data throughout the research process. During the data collection process, strict protocols were followed to maintain data quality and accuracy. We encourage other researchers to use our data for further exploration and verification of the results presented in this article, to promote scientific progress and knowledge sharing in the relevant field. Please feel free to contact us if you have any questions or need further clarification regarding the data availability.

## SUPPLEMENTAL MATERIAL

Supplemental material jvi.01701-24-s0001.docx Figures S1 to S3; Tables S1 to S3.

ASM does not own the copyrights to Supplemental Material that may be linked to, or accessed through, an article. The authors have granted ASM a non-exclusive, world-wide license to publish the Supplemental Material files. Please contact the corresponding author directly for reuse.
