# Supplementary material for: Autophagy promotes p72 degradation and capsid disassembly during the early phase of African swine fever virus infection
Source: J Virol. 2024 Dec 17;99(1):e01701-24. doi: 10.1128/jvi.01701-24 (PMC11784192; doi:10.1128/jvi.01701-24)
Supplement: Supplemental material — Figures S1 to S3; Tables S1 to S3. [file jvi.01701-24-s0001.docx]

**Supplementary data**

**
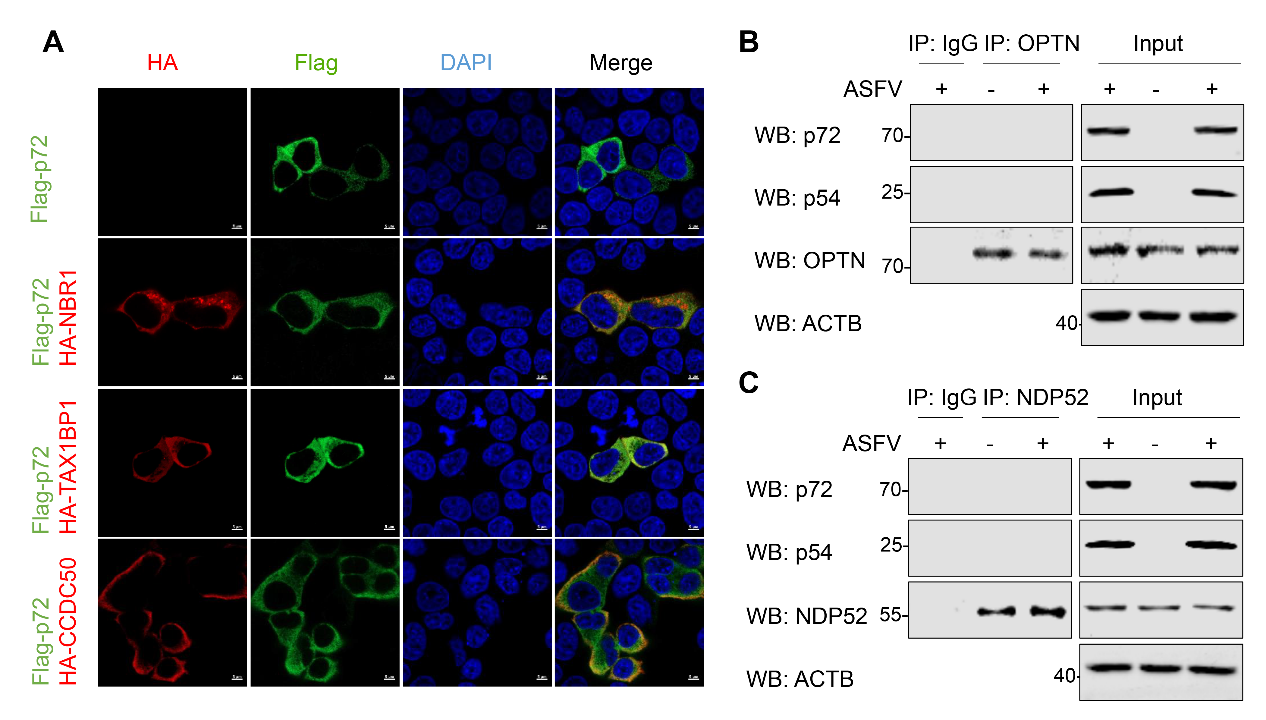
**

**Figure S1. The interaction and colocalization of p72 with autophagy receptors**

**(A)** HEK293T cells were transfected with plasmids expressing FLAG-p72 and HA-tagged autophagy receptors, respectively. The localizations of p72 and autophagy receptors were detected with IFA using anti-FLAG and anti-HA antibodies by confocal microscope. **(B-C)** PAMs were mock-infected or infected with ASFV (MOI = 1) for 36 h, and then Co-IP was performed with anti-OPTN antibody (B) and anti-NDP52 antibody (C). IgG was used as a negative control.

**
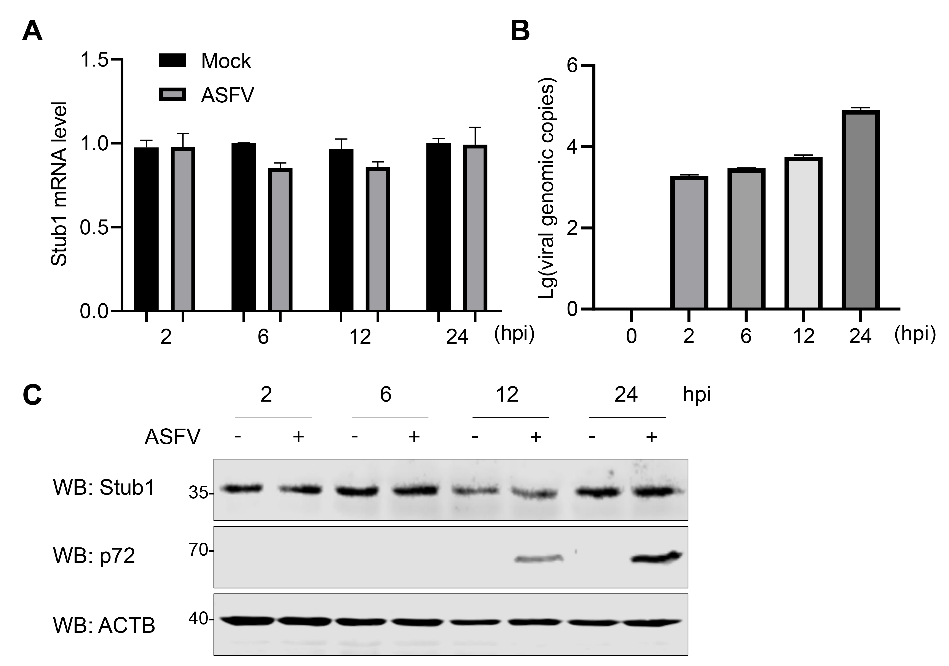
**

**Figure S2. ASFV infection has no effect on the expression of Stub1**

**(A-C)** PAMs were infected with ASFV (MOI = 1) for the indicated time. The cells were lysed, and the mRNA levels of Stub1 (A) and the genome copy numbers of ASFV (B) were determined by qPCR. The expressions of Stub1 were detected by western blotting (C).


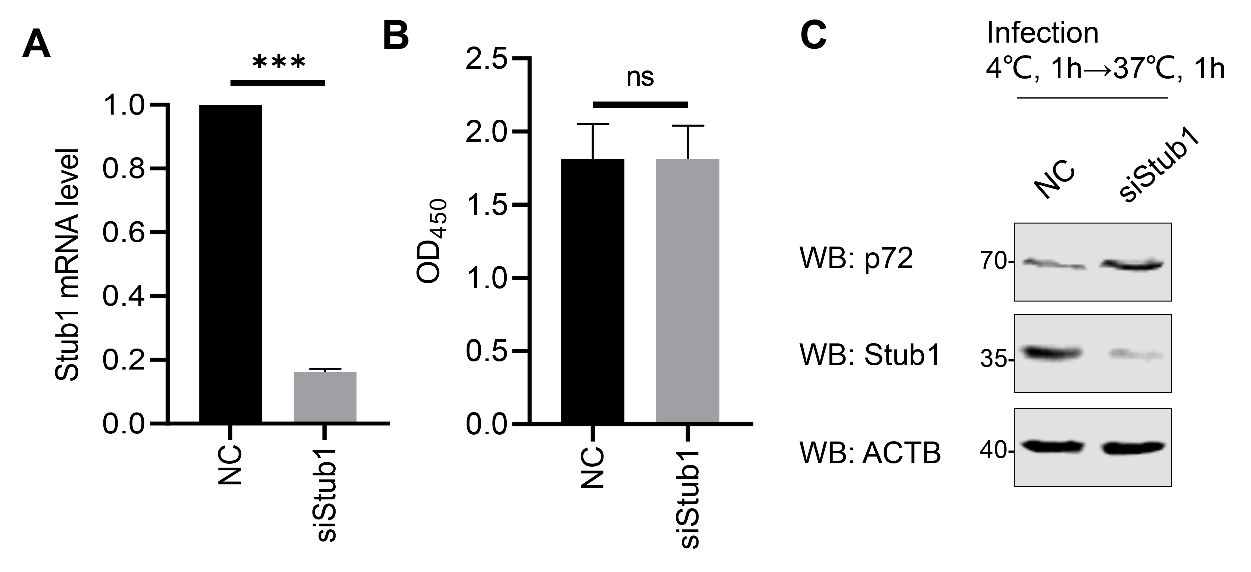


**Figure S3. Knockdown of Stub1 expression inhibits the degradation of p72 during the early phase of ASFV infection**

**(A-B)** PAMs were transfected with siRNAs targeting p62 or LAMP2 for 36 h, and the mRNA levels of Stub1 were determined by qPCR (A), and the cell viability was analyzed using a CCK-8 counting kit (B). **(C)** PAMs were transfected with siRNAs targeting Stub1 for 36 h, and then infected with ASFV (MOI = 10) (4 °C, 1 h, followed by incubation at 37 °C for 1h. The cells were lysed, and the protein lysates were concentrated using TCA precipitation. The expression of p72 and Stub1 was detected using western blotting.

| \| Table S1. Primers and siRNA used in this study. \| \| \| \| \| --- \| --- \| --- \| --- \| \| Resource \| \| Sequence (5’-3’) \| \| \| Primers for qPCR \| Swine Stub1 \| F: TTGCCAACCTGCAGCGAGCCTA \| \| R: CCGCTCCTCGATGCTGTTCCA \| \| ASFV genomic copies \| F: CTGCTCATGGTATCAATCTTATCGA \| \| R: GATACCACAAGATCAGCCGT \| \| siRNA \| Probe: FAM-CCACGGGAGGAATACCAACCCAGTG-TAMRA \| \| Targeting swine HSPA8 \| GCTGTTGTCCAGTCTGATA \| \| Targeting swine Stub1 \| GCAAGGACATTGAGGAGCA \| \| Targeting swine p62 \| GGAACAGATGGAGTCGGAT \| \| Targeting swine LAMP2 \| GCCACTTGCCTTTATGCAA \|   **Table S2. Antibodies used in this study.** | | | |
| --- | --- | --- | --- | --- | --- | --- | --- | --- | --- | --- | --- | --- | --- | --- | --- | --- | --- | --- | --- | --- | --- | --- | --- | --- | --- | --- | --- | --- |
| **Recourse** | **Antibody** | **Company** | **Catalog Number** |
| **Rabbit** | Anti-HA-Tag | Cell Signaling Technology | #3724 |
|  | Anti-RAB5 |  | 3547S |
|  | Anti-RAB7 |  | 9367S |
|  | Anti-FLAG-Tag | Sigma-Aldrich | SAB4301135 |
|  | Anti-MYC-Tag | Proteintech | 16286-1-AP |
|  | Anti-LC3 |  | 14600-1-AP |
|  | Anti-HSC70 |  | 10654-1-AP |
|  | Anti-STUB1 |  | #2080 |
|  | Anti-SQSTM1/p62 |  | 18420-1-AP |
|  | Anti-ubiquitin |  | 10201-2-AP |
|  | Anti-OPTN |  | 10837-1-AP |
|  | Anti-NDP52 |  | 12229-1-AP |
| **Mouse** | Anti-SQSTM1/p62 |  | 66814-1-Ig |
|  | Anti-beta-actin |  | 66009-1-Ig |
|  | Anti-LAMP2 |  | 66301-1-Ig |

| **Table S3. Identification of p72 binding partners by mass spectrometry (1).** | | | | | | | | |
| --- | --- | --- | --- | --- | --- | --- | --- | --- |
| **Number** | **Mass** | | **Score** | **Matches** | **Sequences** | **emPAI** | **Coverage** | **Protein description** |
| 1 | 38864 | 898 | | 104(43) | 9(5) | 1.09 | 28% | CD1d antigen |
| 2 | 38284 | 177 | | 9(5) | 4(3) | 0.28 | 11% | guanine nucleotide-binding protein subunit beta-4 |
| 3 | 33162 | 175 | | 18(7) | 6(4) | 0.61 | 17% | ADP/ATP translocase 2 |
| 4 | 35511 | 169 | | 13(6) | 8(3) | 0.31 | 27% | G-beta like protein |
| 5 | 33117 | 156 | | 12(5) | 6(4) | 0.47 | 17% | mitochondrial solute carrier family 25-member 6 |
| 6 | 35427 | 152 | | 12(5) | 7(5) | 0.56 | 23% | **E3 ubiquitin-protein ligase CHIP (also named Stub1)** |
| 7 | 36717 | 150 | | 19(6) | 5(2) | 0.19 | 13% | Golgi to ER traffic protein 4 homolog isoform X1 |
| 8 | 40420 | 142 | | 9(6) | 4(4) | 0.37 | 8% | solute carrier family 25 member 3 |
| 9 | 34115 | 103 | | 12(5) | 9(4) | 0.59 | 28% | cell division cycle 2 |
| 10 | 32086 | 101 | | 10(3) | 5(3) | 0.34 | 14% | voltage-dependent anion channel 2 |
| 11 | 34904 | 97 | | 12(4) | 9(3) | 0.31 | 24% | mitochondrial glutamate carrier 1 |
| 12 | 70340 | 95 | | 10(3) | 6(2) | 0.10 | 10% | heat shock protein 70.2 |
| 13 | 36283 | 84 | | 6(3) | 4(2) | 0.19 | 12% | ELAV (embryonic lethal, abnormal vision, Drosophila)-like 1 |
| 14 | 32237 | 82 | | 14(6) | 7(4) | 0.63 | 14% | serine/threonine-protein phosphatase PGAM5, mitochondrial isoform X1 |
| 15 | 30148 | 76 | | 8(3) | 7(3) | 0.52 | 23% | ribosomal protein L7a |
| 16 | 12823 | 72 | | 8(1) | 5(1) | 0.60 | 26% | histone H1.3-like protein, partial |
| 17 | 16212 | 66 | | 6(3) | 4(2) | 0.46 | 30% | beta-globin |
| 18 | 25433 | 65 | | 3(1) | 2(1) | 0.13 | 6% | SFRS2 |
| 19 | 36169 | 63 | | 3(1) | 3(1) | 0.19 | 9% | annexin A5 |
| 20 | 32352 | 61 | | 4(1) | 2(1) | 0.10 | 8% | malectin |
| 21 | 29102 | 54 | | 9(4) | 8(4) | 0.72 | 27% | proliferating cell nuclear antigen |
| 22 | 30529 | 50 | | 3(2) | 2(2) | 0.23 | 6% | acidic leucine-rich nuclear phosphoprotein 32 family member E |
| 23 | 26903 | 50 | | 3(1) | 3(1) | 0.42 | 13% | EF-hand domain-containing protein D1 isoform X1 |

**Reference**

1. Chen X, Zheng J, Liu C, Li T, Wang X, Li X, Bao M, Li J, Huang L, Zhang Z, Bu Z, Weng C. 2023. CD1d facilitates African swine fever virus entry into the host cells via clathrin-mediated endocytosis. Emerg Microbes Infect doi:10.1080/22221751.2023.2220575:2220575.
